# Risk of epilepsy in opposite-sex and same-sex twins: a twin cohort study

**DOI:** 10.1186/s13293-018-0179-5

**Published:** 2018-06-04

**Authors:** Yanyan Mao, Linda Juel Ahrenfeldt, Kaare Christensen, Chunsen Wu, Jakob Christensen, Jørn Olsen, Yuelian Sun

**Affiliations:** 1 School of Public Health, Fudan University, Shanghai, China; 2 Key Lab. of Reproduction Regulation of NPFPC, SIPPR, IRD, Fudan University, Shanghai, China; 3 Unit of Epidemiology, Biostatistics and Biodemography, Department of Public Health, University of Southern Denmark, Odense, Denmark; 4 Research Unit of Gynecology and Obstetrics, Institute of Clinical Research, University of Southern Denmark, Odense, Denmark; 5 Department of Obstetrics and Gynecology, Odense University Hospital, Odense, Denmark; 6 Department of Neurology, Institute for Clinical Medicine, Aarhus University Hospital, Aarhus, Denmark; 7 Department of Clinical Epidemiology, Institute for Clinical Medicine, Aarhus University Hospital, Olof Palmes Allé 43-45, DK-8200 Aarhus N, Denmark; 8 Department of Epidemiology, Fielding School of Public Health, University of California, Los Angeles (UCLA), California, LA USA

**Keywords:** Twins, Opposite-sex, Same-sex, Sex difference, Testosterone, Epilepsy

## Abstract

**Background:**

There is a complex interaction between female and male sex hormones and the risk of epilepsy. Whether prenatal exposure to higher levels of sex hormones affects the development of epilepsy in childhood or later in life is not well known. The sex hormone environment of fetuses may be affected by the sex of the co-twin. We estimated the risk of epilepsy for twins with an opposite-sex (OS) co-twin compared with twins with a same-sex (SS) co-twin.

**Methods:**

From the Danish Twin Registry, we identified OS female twins (*n* = 11,078), SS female twins (*n* = 19,186), OS male twins (*n* = 11,080), and SS male twins (*n* = 20,207) born between 1977 and 2009. The SS twins include monozygotic twins, dizygotic twins, and twins with unknown zygosity. These children were followed up from day 29 after birth until diagnosis of epilepsy, death, emigration, or end of follow-up (31 December 2011) whichever came first. Information on diagnosis of epilepsy was obtained from the Danish National Patient Registry. We calculated hazard ratios (HRs) and 95% confidence intervals (CIs) for epilepsy in the OS twins using a Cox proportional hazards regression model compared with the SS twins. To account for the correlation of twins from the same mother when estimating standard errors, we used the cluster option in Stata.

**Results:**

We identified 152 OS female twins, 282 SS female twins, 162 OS male twins, and 335 SS male twins diagnosed with epilepsy corresponding to an incidence rate of 9.9 and 9.7 per 10,000 person years for the OS and SS female twins, and 10.6 and 10.9 per 10,000 person years for the OS and SS male twins, respectively. We found a similar risk of epilepsy among the OS and SS female twins [HR = 1.01; 95% CI 0.83–1.24] as well as among the OS and SS male twins [HR = 0.94; 95% CI 0.78–1.14]

**Conclusions:**

In this population-based study of Danish twins, we did not find difference in the risk of epilepsy between twins with an OS co-twin and twins with a SS co-twin. This applied to both female and male twins. The study therefore does not support the hypothesis that subtle hormone difference in fetal life due to co-twin may play a role in the development of epilepsy later in life.

## Background

Epilepsy is the most frequent chronic neurologic condition in childhood [1–3]. The incidence rate is highest during infancy [3], and males and females have different susceptibility to epilepsy in different periods of life [4]. Females have a higher incidence rate of epilepsy than males in their teenage years, while the rate is higher in males from birth to the beginning of adolescence [4]. On average, males have a marginally higher incidence of epilepsy than females while generalized epilepsies are more common among females and epilepsy with focal seizure are more common among males [5]. Epilepsy has a high degree of heritability indicating shared genetic components in epileptogenesis [6–8], but intrauterine factors like maternal preeclampsia [9], infections [10], and medication use in pregnancy [11] have also been associated with risk of epilepsy. Whether prenatal exposure to subtle differences in sex hormones plays a role in the development of epilepsy remains uncertain [12].

The fetal testes start to produce testosterone in about gestational week 8 [13, 14], and testosterone is an important hormone in male fetal development. It can pass the placenta barrier and the blood-brain barrier [15, 16] and influence early human brain development [17]. The twin testosterone transfer (TTT) hypothesis [18, 19] states that testosterone from a male fetus can be transferred to an adjacent fetus via amniotic diffusion. The opposite-sex (OS) twins therefore have an intrauterine environment that differs from that of the same-sex (SS) twins concerning sex hormones. Thus, OS female twins may be exposed to higher levels of testosterone in utero than SS females [15, 20]. Likewise, SS male twins may have slightly higher prenatal exposure to testosterone compared with OS male twins [21]. Some studies have shown that OS female twins have masculinization of a variety of traits [18, 22], and OS male twins have de-masculinized features compared with SS male twins [23, 24]. A number of studies, however, did not find any notable difference among OS and SS twins regarding academic performance [25] and cancer risk [26]. No clear difference was found regarding early life mortality risks either [27].

Some studies indicate that testosterone exposure in pregnancy might influence the neuropsychiatric development in children including effects on language [28], emotion [29], and gender-related behavior [13] and may cause disruptive behavior disorders [30] and autistic disorders [31, 32]. For example, a study showed the SS male twins had a higher risk of autism compared with OS male twins [33]. Other studies, however, did not support the TTT hypothesis [34, 35]. Instead, the studies found that girls with a male co-twin had less ADHD and autistic traits than girls with a female co-twin [34, 35] indicating the complexity of the effect of fetal hormones on brain development. The interaction between sex hormones and epilepsy is complex [36]. Progesterone and its metabolites are anticonvulsant and estrogens are proconvulsant in general [36], while testosterone has biphasic effect on seizures according to the levels of its distinct metabolites [12]. A number of sex differences are seen in the brain development that are associated with seizure induction and neonatal males present a greater susceptibility to seizures than neonatal females [37], but it is unclear whether the subtle differences in sex hormone environment (especially testosterone) among twins play a role in the development of epilepsy later in life. The present study investigates the risk of epilepsy in OS and SS twins, assuming that the sex of the co-twin influences the level of prenatal exposure to sex hormones and thus the risk of epilepsy.

## Methods

### Study population and follow-up

**Fig. 1:**
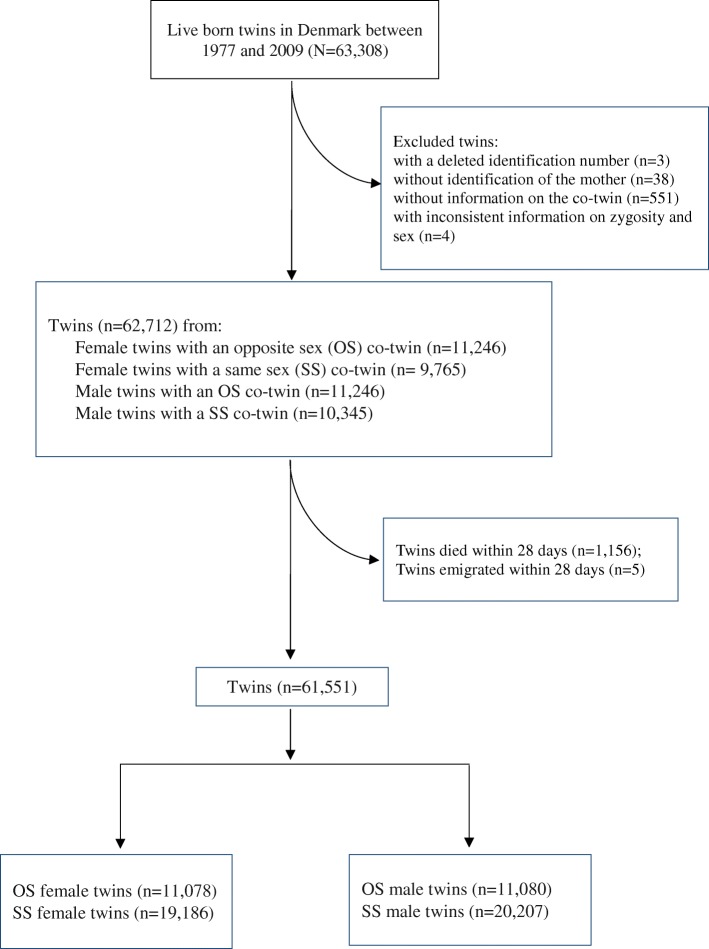
Flowchart of the study population

We identified 63,308 twins born alive in Denmark between 1977 and 2009 from the Danish Twin Registry [38], which has complete registration of all twin live births since 1973. We excluded twins whose identification number was deleted in the registry (*n* = 3), those who were registered without identification of the mother (*n* = 38), those who did not have a recorded twin brother or sister at time of birth (*n* = 551), and those who were registered as monozygotic SS twins but were of opposite sex (*n* = 4). This left us with a study population of 62,712 twins (Fig. 1). In this study population, 1156 (1.8%) children died (329 OS twins and 827 SS twins) and 5 children emigrated within the first 28 days of life, leaving 61,551 twins for the analyses (Fig. 1). These children were followed from day 29 after birth until diagnosis of epilepsy, death, emigration, or end of follow-up (31 December 2011), whichever came first.

The OS twins are dizygotic twins, and the information on the SS twins’ zygosity in the Danish Twin Registry relies on the twins’ answers to a short questionnaire on the degree of similarity between co-twins [39]. Zygosity is not known for all SS twins because of no responses or inconsistent answers to the questionnaire between twins, or death of one or both twins at an early age [39]. Zygosity of SS twins was classified into three groups (monozygotic twins, dizygotic twins, and twins with unknown zygosity) [38].

Information on epilepsy diagnoses was obtained from the Danish National Patient Registry, which contains discharge diagnoses for all inpatients admitted to Danish hospitals from 1977 onwards and for outpatients from 1995 onwards. Diagnoses in this registry were coded according to ICD-8 from 1977 to 1993 and ICD-10 from 1994 onwards. The twins were identified as having epilepsy if they had been hospitalized or been in outpatient care and registered with the ICD-10 code G40-41or the ICD-8 code 345. Information on birth date and parental age at time of birth was obtained from the National Birth Registry [40], and information on parental education was obtained from the Danish Education Registers [41].

### Statistical analyses

We used the Cox proportional hazards regression model to estimate hazard ratios (HRs) and 95% confidence intervals (CIs) of epilepsy for the OS male and female twins (vs the SS twins). We performed both crude and adjusted HRs of epilepsy. The adjusted analyses were controlled for maternal age at time of birth (< 25, 25–29, 30–34, 35–39, 40+ years), paternal age at birth (< 30, 30–39, 40+ years), the highest degree of education completed by the parents (primary and lower secondary, upper and post-secondary, and tertiary), and calendar years (1977–1984, 1985–1994, 1995–1999, 2000–2004, 2005–2009). We also estimated the age-specific risk of epilepsy (< 1 year, 1–4 years, 5–18 years, and 19–36 years). In a sensitivity analysis, we estimated HRs of epilepsy for the OS twins compared with the dizygotic SS twins only.

Among 61,551 twins from 31,028 mothers, there were 30,605 (98.6%) mothers with one twin pregnancy, 420 (1.4%) mothers with two twin pregnancies, and 3 with three twin pregnancies. In the analyses, both twins from a SS twin pair were included as the reference. To account for the correlations of twins in a pair and siblings of the same mother, we used the cluster option in Stata, which takes correlation of twins from the same mother into consideration for estimating standard error. We also randomly divided the SS twin pairs into two groups and used each of them as the reference in the sensitivity analyses to avoid dependence between the two SS twins from the same twin pair.

## Results

In the study population of 61,551 twins, we identified 22,158 (36.0%) OS twins, including 11,078 OS female twins and 11,080 OS male twins, and 39,393 (64.0%) SS twins including 19,186 SS female twins and 20,207 SS male twins (Fig. 1). Among the SS twins, 6861 (17.4%) were monozygotic twins and 11,749 (29.8%) were dizygotic twins while 20,783 (52.8%) were of unknown zygosity and the distribution was same among the female and male twins (Table 1). In general, the OS female and male twins were more likely born to older parents and born in the later part of the study period (Table 1).

**Table 1:** Characteristics of the study population

	Female twins		Male twins	
Characteristics	OS^a^ (n=11,078)	SS^a^ (n=19,186)	OS^a^ (n=11,080)	SS^a^ (n=20,207)
	N (%)	N (%)	N (%)	N (%)
Zygosity				
Monozygotic	.	3,425 (17.9)	.	3,436 (17.0)
Dizygotic	11,078 (100)	5,719 (29.8)	11,080 (100)	6,030 (29.8)
Unknown	.	10,042 (52.3)	.	10,741 (53.2)
Maternal age at birth (years)				
<25	976 (8.8)	2,662 (13.9)	985 (8.9)	2,727 (13.5)
25-29	3,364 (30.4)	6,514 (34.0)	3,353 (30.3)	6,745 (33.4)
30-35	4,421 (39.9)	6,828 (35.6)	4,421 (39.9)	7,357 (36.4)
35-39	2,029 (18.3)	2,796 (14.6)	2,033 (18.3)	3,021 (15.0)
40+	288 (2.6)	386 (2.0)	288 (2.6)	357 (1.8)
Paternal age at birth (years)				
<30	2,727 (24.6)	5,891 (30.7)	2,728 (24.6)	6,398 (31.7)
30-39	6,883 (62.1)	11,088 (57.8)	6,880 (62.1)	11,468 (56.8)
40+	1,374 (12.4)	2,032 (10.6)	1,375 (12.4)	2,091 (10.3)
Missing	94 (0.8)	175 (0.9)	97 (0.9)	250 (1.2)
Maternal completed highest education				
Primary and lower secondary	1,902 (17.2)	3,600 (18.8)	1,914 (17.3)	3,763 (18.6)
Upper and post-secondary	4,477 (40.4)	7,801 (40.7)	4,468 (40.3)	8,210 (40.6)
Tertiary	4,560 (41.2)	7,476 (39.0)	4,558 (41.1)	7,979 (39.5)
Missing	139 (1.3)	309 (1.6)	140 (1.3)	255 (1.3)
Paternal completed highest education				
Primary and lower secondary	2,038 (18.4)	3,612 (18.8)	2,029 (18.3)	3,909 (19.3)
Upper and post-secondary	5,218 (47.1)	9,140 (47.6)	5,231 (47.2)	9,485 (46.9)
Tertiary	3,561 (32.1)	5,867 (30.6)	3,557 (32.1)	6,190 (30.6)
Missing	261 (2.4)	567 (3.0)	263 (2.4)	623 (3.1)
Calendar year of birth				
1977-1984	1,234 (11.1)	2,806 (14.6)	1,231 (11.1)	3,249 (16.1)
1985-1994	2,451 (22.1)	4,994 (26.0)	2,463 (22.2)	4,953 (24.5)
1995-1999	2,212 (20.0)	3,414 (17.8)	2,213 (20.0)	3,768 (18.6)
2000-2004	2,553 (23.0)	3,913 (20.4)	2,550 (23.0)	4,114 (20.4)
2005-2009	2,628 (23.7)	4,059 (21.2)	2,623 (23.7)	4,123 (20.4)

^a^*OS* opposite-sex twins, i.e., twins with an opposite sex co-twin, *SS* same-sex twins, i.e., twins with a same sex co-twin

The twins were followed for up to 36 years with a median of 13.3 years. During this follow-up, we identified 152 OS female twins, 282 SS female twins, 162 OS male twins, and 335 SS male twins diagnosed with epilepsy corresponding to an incidence rate of 9.9 and 9.7 per 10,000 person years for the OS and SS female twins, and 10.6 and 10.9 per 10,000 person years for the OS and SS male twins, respectively (Table 2).

**Table 2:** Hazard ratios (HR) for epilepsy in OS twins compared with SS twins in females and males

Exposure status	Both twins of the SS twin pair were included in the reference					One twin of the SS twin pair was randomly selected as the reference	
						Sample 1	Sample 2
	Population	Epilepsy cases	IR^a^	Crude HR	Adjusted HR [95% CI]^b^	Adjusted HR [95% CI]^b^	Adjusted HR [95% CI]^b^
The SS female twins	19,186	282	9.74	Ref	Ref	Ref	Ref
The OS female twins	11,078	152	9.91	1.00	1.01[0.83-1.24]	1.03[0.81-1.30]	1.00[0.79-1.25]
The SS male twins	20,207	335	10.9	Ref	Ref	Ref	Ref
The OS male twins	11,080	162	10.6	0.93	0.94[0.78-1.14]	1.00[0.80-1.24]	0.89[0.72-1.11]

^a^Incidence rate (/10,000 person-years)

^b^Hazard ratios are adjusted for maternal age at time of birth (<25, 25-29, 30-34, 35-39, 40+ years), paternal age at time of birth (<30, 30-39, 40+ years, missing), maternal and paternal highest completed education (primary and lower secondary, upper and post-secondary, tertiary, and missing), and calendar year (1977-1984, 1985-1994, 1995-1999, 2000-2004, 2005-2009)

Overall, there was no significant difference in the risk of epilepsy between OS and SS female twins or between OS and SS male twins. The adjusted HR of epilepsy was 1.01 [95% CI 0.83–1.24] for the OS compared with SS female twins and 0.94 [95% CI 0.78–1.14] for the OS compared with SS male twins (Table 2). The results remained unchanged when we repeated the analyses using the random samples of SS twins as the reference group (Table 2). The adjusted HR of epilepsy was 1.02 [95% CI 0.79–1.32] for OS vs SS females twins and 0.94 [95% CI 0.73–1.22] for OS vs SS males twins when we did the analyses among the dizygotic twins only (data not shown in the tables). When we estimated the age-specific risk of epilepsy, the results suggested that OS female twins had a decreased risk of epilepsy in the first year of life [HR 0.74; 95% CI 0.42–1.30] compared with SS female twins, but the 95% CI was wide and covered one (Table 3). The OS male twins had a similar risk of epilepsy in the first year of life as the SS male twins [HR 0.98; 95% CI 0.63–1.51] while they had a lower risk of epilepsy in early adulthood although the result was non-significant (Table 3).

**Table 3:** Age specific hazard ratios (HR) for epilepsy in OS twins compared with SS twins

Age group and exposure status	Female					Males				
	Population	Epilepsy cases	IR^a^	Crude HR	Adjusted HR [95% CI]^b^	Population	Epilepsy cases	IR^a^	Crude HR	Adjusted HR [95% CI]^b^
<1 year										
SS	19,186	47	26.66	Ref	Ref	20,207	63	33.99	Ref	Ref
OS	11,078	19	18.65	0.70	0.74[0.42-1.30]	11,080	34	33.40	0.98	0.98[0.63-1.51]
1-9 years										
SS	19,039	130	9.04	Ref	Ref	19,990	176	11.60	Ref	Ref
OS	11,004	86	10.55	1.16	1.17[0.89-1.55]	10,992	87	10.69	0.92	0.95[0.73-1.23]
10-18 years										
SS	12,379	73	8.81	Ref	Ref	13,150	70	8.01	Ref	Ref
OS	6,722	35	8.38	0.95	0.95[0.63-1.44]	6,713	34	8.16	1.01	0.98[0.65-1.48]
19-35 years										
SS	6,282	32	7.06	Ref	Ref	6,533	26	5.19	Ref	Ref
OS	2,805	12	6.05	0.86	0.87[0.45-1.69]	2,789	7	3.58	0.69	0.72[0.31-1.66]

^a^Incidence rate (/10,000 person-years)

^b^Hazard ratios, adjusted for maternal age at time of birth (<25, 25-29, 30-34, 35-39, 40+ years), paternal age at time of birth (<30, 30-39, 40+ years, missing), maternal and paternal highest completed education (primary and lower secondary, upper and post-secondary, tertiary, and missing), and calendar year (1977-1984, 1985-1994, 1995-1999, 2000-2004, 2005-2009)

## Discussion

In this population-based study of Danish twins, we found that twins who shared the uterus with an OS co-twin had a similar risk of epilepsy as twins with a SS co-twin. The results indicated that the OS female twins had a lower risk of epilepsy in the first year of life compared with the SS female twins but the confidence intervals were wide and inconclusive.

Sex hormones are important for the sexual differentiation of the brain during early development, and testosterone is considered the main hormone responsible for this development. Male fetal testosterone production begins in the first trimester and peaks around gestational week 16 followed by another surge in the perinatal period [37]. Animal studies have shown that testosterone affects sex-specific volumes of brain nuclei [37] and neuronal structure and function [36]. Testosterone may be associated with increased excitatory tone in specific brain areas of neonatal males [37]. When compared to neonatal females, neonatal male rats have an increased baseline neuronal excitability and a more reactive neuroimmune response in brain areas associated with seizure induction [37]. Based on the TTT hypothesis, OS female twins would therefore be expected to a have higher risk of epilepsy or seizure early in life, but we did not find evidence to support this hypothesis. On the contrary, our findings showed that the female twins with an OS male twin had a lower risk of epilepsy in the first year of life compared with the SS female twins although the findings were not statistically significant. We also found the OS and SS male twins had a similar risk of epilepsy.

As far as we know, no previous studies have compared the risk of epilepsy in OS and SS twins. Findings for attention deficit hyperactivity disorder (ADHD) and autism are conflicting [33–35]. Higher levels of testosterone in amniotic fluid have been associated with autism in some [32], but not all studies [42, 43]. Similar to our findings on risk of epilepsy in the first year of life among the OS female twins, recent studies on the risk of ADHD and autistic traits in the OS twins reported opposite findings [34, 35] according to the TTT hypothesis, which is interesting, and more studies are needed to explore the influence of fetal hormone on brain development and neuropsychiatric disorders.

The major strength of this study is the availability of reliable data on epilepsy from a large population-based register for all Danish twins born in 1977–2009 with almost complete follow-up. However, there are also limitations. To estimate the risk of epilepsy among the OS twins, which are dizygotic twins, the ideal reference group is the SS dizygotic twins [21]. The large group of twins with unknown zygosity among the SS twins in our study made it impossible to make the most valid comparison by identifying all the dizygotic SS twins. Moreover, we were not able to control for the use of assisted reproductive technology (ART) or other sources of sex hormone in fetal life. A higher proportion of children born after ART would be expected in the OS than in the SS twin group [44]. Persons who undergo fertility treatments may have conditions [45] that would be associated with the risk of epilepsy in offspring later in life and could potentially bias our findings [46]. In addition, the difference in hormone levels between OS and SS twins might not be large enough to have impact on the development of epilepsy and results from animal studies need not to apply to human. Likewise, testosterone affecting the fetus’ epileptogenesis during pregnancy might be more complex, which cannot be demonstrated in the current study. Animal models have shown that testosterone can be metabolized into estradiol, which is generally excitatory, or it can be metabolized into androstandediol and dihydrotestosterone, which exert potent antiepileptic effect [12]. Due to sex difference in the prevalence of subtypes of epilepsy [5, 47], it is important to explore effects of sex hormones in prenatal life on the development of the subtypes of epilepsy. Unfortunately, we did not have sufficient power to estimate the risk of the subtypes of epilepsy and the validity of the epilepsy subtype diagnosis is low in the Danish National Patient Registry [48].

## Conclusions

In this population-based study of Danish twins, we did not find difference in the risk of epilepsy between twins with an OS co-twin and SS co-twin. This applied to both female and male twins. The study therefore does not support the hypothesis that subtle hormone difference in fetal life due to co-twin may play a role in the development of epilepsy later in life.

## Abbreviations

ADHD: Attention deficit hyperactivity disorder
ART: Assisted reproductive technology
CI: Confidence interval
HR: Hazard ratio
ICD: International Classification of Diseases
OS: Opposite sex
SS: Same sex
TTT: Twin testosterone transfer
